# Prop-2-yn-1-yl 4,6-di-*O*-acetyl-2,3-dide­oxy-α-d-*erythro*-hex-2-enopyran­o­side

**DOI:** 10.1107/S1600536811047246

**Published:** 2011-11-16

**Authors:** Fanuel M. Mesfin, Henok H. Kinfe, Alfred Muller

**Affiliations:** a Research Center for Synthesis and Catalysis, Department of Chemistry, University of Johannesburg (APK Campus), PO Box 524, Auckland Park, Johannesburg 2006, South Africa

## Abstract

The absolute structure of the title compound, C_13_H_16_O_6_, was determined. The pyranosyl ring adopting an envelope conformation. The acetyl groups are located in equatorial positions. The crystal structure features weak C—H⋯O inter­actions.

## Related literature

For details of the Ferrier arrangement, see: Ferrier & Prasad (1969 ▶) and for the synthesis of pseudoglycals utilizing the Ferrier arrangement, see: López *et al.* (1995 ▶); Yadav *et al.* (2001 ▶). For background to the synthetic methodology of glycosides, see: Kinfe *et al.* (2011 ▶); Breton (1997 ▶). For ring puckering analysis, see: Cremer & Pople (1975 ▶).
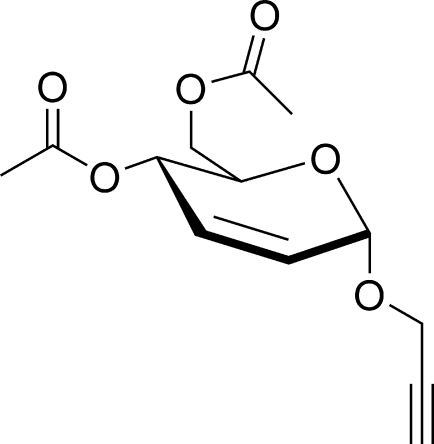


## Experimental

### 

#### Crystal data

C_13_H_16_O_6_
*M* *_r_* = 268.26
Orthorhombic, 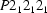

*a* = 5.2277 (2) Å
*b* = 14.8549 (5) Å
*c* = 17.0509 (5) Å
*V* = 1324.12 (8) Å^3^
*Z* = 4
Cu *K*α radiation
μ = 0.91 mm^−1^
*T* = 100 K
0.28 × 0.06 × 0.06 mm

#### Data collection

Bruker APEX DUO 4K CCD diffractometer
Absorption correction: multi-scan (*SADABS*; Bruker, 2008 ▶) *T*_min_ = 0.785, *T*_max_ = 0.948
12479 measured reflections
2184 independent reflections
2113 reflections with *I* > 2σ(*I*)
*R*_int_ = 0.039

#### Refinement

*R*[*F* ^2^ > 2σ(*F* ^2^)] = 0.023
*wR*(*F* ^2^) = 0.058
*S* = 1.06
2184 reflections
174 parameters
H-atom parameters constrained
Δρ_max_ = 0.1 e Å^−3^
Δρ_min_ = −0.12 e Å^−3^
Absolute structure: Flack (1983 ▶), 872 Friedel pairs
Flack parameter: −0.05 (14)

### 

Data collection: *APEX2* (Bruker, 2011 ▶); cell refinement: *SAINT* (Bruker, 2008 ▶); data reduction: *SAINT* and *XPREP* (Bruker, 2008 ▶); program(s) used to solve structure: *SIR97* (Altomare *et al.*, 1999 ▶); program(s) used to refine structure: *SHELXL97* (Sheldrick, 2008 ▶); molecular graphics: *DIAMOND* (Brandenburg & Putz, 2005 ▶); software used to prepare material for publication: *WinGX* (Farrugia, 1999 ▶).

## Supplementary Material

Crystal structure: contains datablock(s) global, I. DOI: 10.1107/S1600536811047246/nc2253sup1.cif

Structure factors: contains datablock(s) I. DOI: 10.1107/S1600536811047246/nc2253Isup2.hkl

Additional supplementary materials:  crystallographic information; 3D view; checkCIF report

## Figures and Tables

**Table 1:** Hydrogen-bond geometry (Å, °)

*D*—H⋯*A*	*D*—H	H⋯*A*	*D*⋯*A*	*D*—H⋯*A*
C8—H8⋯O4^i^	0.95	2.37	3.2139 (17)	149
C11—H11*B*⋯O6^ii^	0.98	2.58	3.4869 (16)	154
C13—H13*C*⋯O6^iii^	0.98	2.38	3.3152 (19)	159

Symmetry codes: (i) 
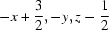
; (ii) 
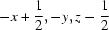
; (iii) 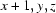.

**Fig. 1.**
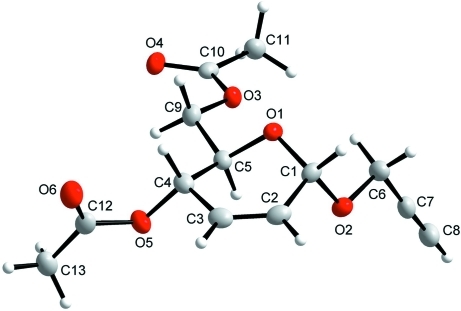
: Crystal structure of the title compound with labelling and displacement ellipsoids drawn at the 50% probability level.

## supplementary crystallographic information

### Comment

Treatment of 3,4,6-tri-*O*-acetyl-D-glucal with a Lewis acid catalyst in the presence of alcohols and other C and S nucleophiles results to allylic rearrangement of the glucal and the reaction is called Ferrier rearrangement (Ferrier & Prasad, 1969; López *et al.*, 1995; Yadav *et al.*, 2001; Kinfe *et al.*, 2011). Recently, we reported the synthesis of the title compound by treating a glucal with NaHSO_4_ supported on silica gel in the presence of propargyl alcohol (Kinfe *et al.*, 2011). Herein, we report the crystal structure of the pure diastereomer obtained by crystallization from the mixture of products.

In the crystal structure of the title compound the acetyl groups are in equatorial positions (see Fig. 1). The pyran ring adopts an envelope conformation with ring puckering parameters of q_2_ = 0.4180 (14) Å, q_3_ = 0.3070 (14) Å, Q = 0.5186 (13) Å and φ_2_ = 324.1 (2)° (see Cremer & Pople, 1975). Several weak C—H···O interactions are noted and listed in Table 1.

### Experimental

To a solution of a tri-*O*-acetyl-D-glucal (100 mg, 0.36 mmol) and propargyl alcohol (0.042 ml, 0.72 mmol) in CH_3_CN (1 ml) NaHSO_4_-SiO_2_ (2.5 mg, 3.0 mmol NaHSO_4_/g) was added (see Breton, 1997). The resulting mixture was stirred at 80 °C for 5 min. After adding silica gel to the reaction mixture at room temperature, the solvent was evaporated *in vacuo* without heating until a free-flowing solid was obtained. The resulting solid was column chromatographed using 1:9 ethyl acetate:hexane eluent to afford α:β (6:1) mixture of 2,3-unsaturated glycosides in 90% yield as a white solid. Recrystalization from a mixture of DCM and hexane afforded the title compound in 50% yield as colorless crystals.

### Refinement

All hydrogen atoms were positioned in geometrically idealized positions with C—H = 1.00 Å (methine), 0.99 Å (methylene), 0.98 Å (methyl) and 0.95 Å (aromatic and acetylenic). All hydrogen atoms were allowed to ride on their parent atoms with *U*_iso_(H) = 1.2*U*_eq_, except for the methyl where *U*_iso_(H) = 1.5*U*_eq_ was utilized. The initial positions of methyl hydrogen atoms were located from a Fourier difference map and refined as fixed rotor. The D enantiomer was determined on the basis of 872 Friedel pairs with the final Flack parameter refined to -0.05 (14). The highest residual electron density of 0.10 e.Å^-3^ is 0.63 Å from H6A representing no physical meaning.

### Crystal data

**Table:**

C_13_H_16_O_6_	*F*(000) = 568
*M**_r_* = 268.26	*D*_x_ = 1.346 Mg m^−^^3^
Orthorhombic, *P*2_1_2_1_2_1_	Cu *K*α radiation, λ = 1.54178 Å
Hall symbol: P 2ac 2ab	Cell parameters from 7170 reflections
*a* = 5.2277 (2) Å	θ = 6.0–64.2°
*b* = 14.8549 (5) Å	µ = 0.91 mm^−^^1^
*c* = 17.0509 (5) Å	*T* = 100 K
*V* = 1324.12 (8) Å^3^	Needle, colourless
*Z* = 4	0.28 × 0.06 × 0.06 mm

### Data collection

**Table:**

Bruker APEX DUO 4K CCD diffractometer	2184 independent reflections
Radiation source: Incoatec IµS microfocus X-ray source	2113 reflections with *I* > 2σ(*I*)
Incoatec Quazar Multilayer Mirror	*R*_int_ = 0.039
Detector resolution: 8.4 pixels mm^-1^	θ_max_ = 64.6°, θ_min_ = 6.0°
φ and ω scans	*h* = −6→5
Absorption correction: multi-scan (*SADABS*; Bruker, 2008)	*k* = −17→17
*T*_min_ = 0.785, *T*_max_ = 0.948	*l* = −19→17
12479 measured reflections	

### Refinement

**Table:**

Refinement on *F*^2^	Secondary atom site location: difference Fourier map
Least-squares matrix: full	Hydrogen site location: inferred from neighbouring sites
*R*[*F*^2^ > 2σ(*F*^2^)] = 0.023	H-atom parameters constrained
*wR*(*F*^2^) = 0.058	*w* = 1/[σ^2^(*F*_o_^2^) + (0.0281*P*)^2^ + 0.1772*P*] where *P* = (*F*_o_^2^ + 2*F*_c_^2^)/3
*S* = 1.06	(Δ/σ)_max_ < 0.001
2184 reflections	Δρ_max_ = 0.1 e Å^−^^3^
174 parameters	Δρ_min_ = −0.12 e Å^−^^3^
0 restraints	Absolute structure: Flack (1983), 872 Friedel pairs
Primary atom site location: structure-invariant direct methods	Flack parameter: −0.05 (14)

### Special details

**Table:**

Experimental. The intensity data was collected on a Bruker Apex DUO 4 K CCD diffractometer using an exposure time of 5 s/frame. A total of 2276 frames were collected with a frame width of 1° covering up to θ = 64.63° with 98.1% completeness accomplished.Analytical data: ^1^H NMR (CDCl_3_, 300 MHz): δ 5.90 (d, *J* = 10.4 Hz, 1H), 5.82 (td, *J* = 2.4 and 10.0 Hz, 1H), 5.32 (dd, *J* = 1.2 and 9.6 Hz, 1H), 5.21 (s, 1H), 4.40–4.03 (m, 5H), 2.44 (t, *J* = 2.4 Hz, 1H), 2.08 (s, 3H), 2.07 (s, 3H); ^13^C NMR (CDCl_3_, 75 MHz): δ 170.8, 170.2, 129.8, 127.2, 92.8, 79.0, 74.8, 67.2, 65.1, 62.7, 55.0, 20.9, 20.8
Geometry. All e.s.d.'s (except the e.s.d. in the dihedral angle between two l.s. planes) are estimated using the full covariance matrix. The cell e.s.d.'s are taken into account individually in the estimation of e.s.d.'s in distances, angles and torsion angles; correlations between e.s.d.'s in cell parameters are only used when they are defined by crystal symmetry. An approximate (isotropic) treatment of cell e.s.d.'s is used for estimating e.s.d.'s involving l.s. planes.
Refinement. Refinement of *F*^2^ against ALL reflections. The weighted *R*-factor *wR* and goodness of fit *S* are based on *F*^2^, conventional *R*-factors *R* are based on *F*, with *F* set to zero for negative *F*^2^. The threshold expression of *F*^2^ > σ(*F*^2^) is used only for calculating *R*-factors(gt) *etc*. and is not relevant to the choice of reflections for refinement. *R*-factors based on *F*^2^ are statistically about twice as large as those based on *F*, and *R*- factors based on ALL data will be even larger.

### Fractional atomic coordinates and isotropic or equivalent isotropic displacement parameters (Å^2^)

**Table:**

	*x*	*y*	*z*	*U*_iso_*/*U*_eq_	
C1	−0.1501 (2)	0.08128 (8)	0.42326 (7)	0.0220 (3)	
H1	−0.2982	0.0577	0.3924	0.026*	
C2	−0.2436 (3)	0.15417 (9)	0.47693 (8)	0.0249 (3)	
H2	−0.3473	0.201	0.4562	0.03*	
C3	−0.1854 (3)	0.15485 (9)	0.55205 (8)	0.0255 (3)	
H3	−0.2594	0.1994	0.585	0.031*	
C4	−0.0052 (2)	0.08713 (9)	0.58703 (7)	0.0229 (3)	
H4	−0.1016	0.0424	0.6194	0.028*	
C5	0.1419 (3)	0.03970 (8)	0.52242 (7)	0.0204 (3)	
H5	0.2635	0.083	0.4974	0.024*	
C6	0.1114 (3)	0.05767 (9)	0.31257 (7)	0.0268 (3)	
H6A	−0.0353	0.0393	0.2795	0.032*	
H6B	0.1814	0.0032	0.3383	0.032*	
C7	0.3070 (3)	0.09936 (8)	0.26419 (7)	0.0256 (3)	
C8	0.4705 (3)	0.12937 (9)	0.22357 (8)	0.0312 (3)	
H8	0.6014	0.1534	0.1911	0.037*	
C9	0.2870 (3)	−0.04105 (9)	0.55155 (7)	0.0229 (3)	
H9A	0.4102	−0.0229	0.5927	0.028*	
H9B	0.1674	−0.0857	0.5742	0.028*	
C10	0.6141 (2)	−0.13598 (8)	0.50283 (7)	0.0201 (3)	
C11	0.7222 (3)	−0.17820 (9)	0.43096 (8)	0.0269 (3)	
H11A	0.9074	−0.1854	0.4371	0.04*	
H11B	0.6871	−0.1397	0.3856	0.04*	
H11C	0.6432	−0.2373	0.4229	0.04*	
C12	0.1406 (3)	0.13903 (8)	0.71258 (7)	0.0235 (3)	
C13	0.3572 (3)	0.18527 (9)	0.75213 (8)	0.0292 (3)	
H13A	0.3149	0.195	0.8075	0.044*	
H13B	0.3885	0.2434	0.7267	0.044*	
H13C	0.5111	0.1479	0.7483	0.044*	
O1	−0.03843 (18)	0.00939 (5)	0.46538 (5)	0.0208 (2)	
O2	0.02791 (19)	0.12087 (6)	0.37098 (5)	0.0234 (2)	
O3	0.42076 (17)	−0.07942 (6)	0.48563 (5)	0.0217 (2)	
O4	0.68222 (18)	−0.15169 (6)	0.56925 (5)	0.0254 (2)	
O5	0.18437 (19)	0.13295 (6)	0.63443 (5)	0.0272 (2)	
O6	−0.0477 (2)	0.10886 (6)	0.74305 (5)	0.0322 (2)	

### Atomic displacement parameters (Å^2^)

**Table:**

	*U*^11^	*U*^22^	*U*^33^	*U*^12^	*U*^13^	*U*^23^
C1	0.0193 (7)	0.0225 (6)	0.0240 (6)	0.0010 (6)	−0.0010 (5)	0.0055 (5)
C2	0.0188 (7)	0.0219 (6)	0.0339 (7)	0.0009 (5)	0.0051 (6)	0.0042 (6)
C3	0.0203 (7)	0.0245 (7)	0.0318 (7)	−0.0026 (6)	0.0075 (6)	−0.0021 (5)
C4	0.0200 (7)	0.0265 (7)	0.0223 (6)	−0.0051 (6)	0.0034 (5)	−0.0019 (5)
C5	0.0194 (7)	0.0227 (6)	0.0191 (6)	−0.0035 (5)	0.0003 (5)	0.0003 (5)
C6	0.0343 (8)	0.0235 (7)	0.0226 (6)	−0.0009 (6)	0.0030 (6)	−0.0016 (5)
C7	0.0319 (8)	0.0258 (7)	0.0191 (6)	0.0024 (6)	−0.0035 (6)	−0.0030 (5)
C8	0.0379 (9)	0.0341 (7)	0.0217 (6)	−0.0062 (7)	0.0024 (6)	−0.0026 (6)
C9	0.0220 (7)	0.0276 (7)	0.0192 (6)	−0.0005 (5)	0.0015 (5)	0.0016 (5)
C10	0.0183 (7)	0.0172 (6)	0.0248 (7)	−0.0032 (5)	−0.0028 (5)	0.0028 (5)
C11	0.0307 (8)	0.0226 (6)	0.0273 (7)	0.0037 (6)	−0.0025 (6)	−0.0012 (5)
C12	0.0249 (8)	0.0231 (6)	0.0225 (6)	0.0075 (5)	0.0029 (6)	−0.0013 (5)
C13	0.0300 (9)	0.0310 (7)	0.0266 (7)	0.0057 (6)	−0.0026 (6)	−0.0050 (5)
O1	0.0210 (5)	0.0191 (4)	0.0223 (4)	−0.0017 (4)	−0.0029 (4)	0.0018 (3)
O2	0.0266 (5)	0.0209 (4)	0.0227 (4)	0.0002 (4)	0.0046 (4)	0.0021 (3)
O3	0.0220 (5)	0.0246 (4)	0.0184 (4)	0.0029 (4)	−0.0016 (4)	0.0003 (3)
O4	0.0262 (5)	0.0279 (5)	0.0221 (5)	0.0007 (4)	−0.0051 (4)	0.0053 (4)
O5	0.0267 (5)	0.0342 (5)	0.0207 (4)	−0.0084 (4)	0.0034 (4)	−0.0048 (4)
O6	0.0293 (6)	0.0422 (6)	0.0252 (5)	0.0008 (5)	0.0070 (4)	−0.0011 (4)

### Geometric parameters (Å, °)

**Table:**

C1—O1	1.4130 (14)	C7—C8	1.187 (2)
C1—O2	1.4165 (15)	C8—H8	0.95
C1—C2	1.4996 (18)	C9—O3	1.4412 (15)
C1—H1	1	C9—H9A	0.99
C2—C3	1.3164 (19)	C9—H9B	0.99
C2—H2	0.95	C10—O4	1.2100 (15)
C3—C4	1.5018 (18)	C10—O3	1.3465 (15)
C3—H3	0.95	C10—C11	1.4881 (18)
C4—O5	1.4487 (15)	C11—H11A	0.98
C4—C5	1.5170 (17)	C11—H11B	0.98
C4—H4	1	C11—H11C	0.98
C5—O1	1.4272 (15)	C12—O6	1.1999 (17)
C5—C9	1.5038 (18)	C12—O5	1.3550 (15)
C5—H5	1	C12—C13	1.486 (2)
C6—O2	1.4366 (15)	C13—H13A	0.98
C6—C7	1.453 (2)	C13—H13B	0.98
C6—H6A	0.99	C13—H13C	0.98
C6—H6B	0.99		
			
O1—C1—O2	111.24 (10)	C8—C7—C6	176.82 (14)
O1—C1—C2	111.73 (10)	C7—C8—H8	180
O2—C1—C2	107.36 (10)	O3—C9—C5	107.62 (10)
O1—C1—H1	108.8	O3—C9—H9A	110.2
O2—C1—H1	108.8	C5—C9—H9A	110.2
C2—C1—H1	108.8	O3—C9—H9B	110.2
C3—C2—C1	121.60 (12)	C5—C9—H9B	110.2
C3—C2—H2	119.2	H9A—C9—H9B	108.5
C1—C2—H2	119.2	O4—C10—O3	123.03 (11)
C2—C3—C4	121.74 (12)	O4—C10—C11	125.29 (12)
C2—C3—H3	119.1	O3—C10—C11	111.64 (10)
C4—C3—H3	119.1	C10—C11—H11A	109.5
O5—C4—C3	109.64 (10)	C10—C11—H11B	109.5
O5—C4—C5	106.06 (10)	H11A—C11—H11B	109.5
C3—C4—C5	109.91 (10)	C10—C11—H11C	109.5
O5—C4—H4	110.4	H11A—C11—H11C	109.5
C3—C4—H4	110.4	H11B—C11—H11C	109.5
C5—C4—H4	110.4	O6—C12—O5	122.65 (12)
O1—C5—C9	107.86 (10)	O6—C12—C13	126.95 (12)
O1—C5—C4	107.87 (10)	O5—C12—C13	110.39 (12)
C9—C5—C4	112.72 (10)	C12—C13—H13A	109.5
O1—C5—H5	109.4	C12—C13—H13B	109.5
C9—C5—H5	109.4	H13A—C13—H13B	109.5
C4—C5—H5	109.4	C12—C13—H13C	109.5
O2—C6—C7	109.20 (11)	H13A—C13—H13C	109.5
O2—C6—H6A	109.8	H13B—C13—H13C	109.5
C7—C6—H6A	109.8	C1—O1—C5	112.37 (9)
O2—C6—H6B	109.8	C1—O2—C6	111.37 (10)
C7—C6—H6B	109.8	C10—O3—C9	116.18 (9)
H6A—C6—H6B	108.3	C12—O5—C4	117.67 (10)
			
O1—C1—C2—C3	10.49 (18)	C9—C5—O1—C1	−168.46 (10)
O2—C1—C2—C3	−111.74 (14)	C4—C5—O1—C1	69.51 (12)
C1—C2—C3—C4	5.0 (2)	O1—C1—O2—C6	62.63 (13)
C2—C3—C4—O5	131.20 (13)	C2—C1—O2—C6	−174.84 (10)
C2—C3—C4—C5	14.99 (17)	C7—C6—O2—C1	−175.81 (11)
O5—C4—C5—O1	−168.20 (9)	O4—C10—O3—C9	−3.13 (16)
C3—C4—C5—O1	−49.76 (13)	C11—C10—O3—C9	174.60 (11)
O5—C4—C5—C9	72.84 (13)	C5—C9—O3—C10	162.29 (10)
C3—C4—C5—C9	−168.72 (11)	O6—C12—O5—C4	−0.61 (17)
O1—C5—C9—O3	61.81 (13)	C13—C12—O5—C4	178.21 (10)
C4—C5—C9—O3	−179.22 (10)	C3—C4—O5—C12	95.77 (13)
O2—C1—O1—C5	71.48 (13)	C5—C4—O5—C12	−145.60 (10)
C2—C1—O1—C5	−48.49 (14)		

### Hydrogen-bond geometry (Å, °)

**Table:**

*D*—H···*A*	*D*—H	H···*A*	*D*···*A*	*D*—H···*A*
C8—H8···O4^i^	0.95	2.37	3.2139 (17)	149.
C11—H11B···O6^ii^	0.98	2.58	3.4869 (16)	154.
C13—H13C···O6^iii^	0.98	2.38	3.3152 (19)	159.

Symmetry codes: (i) −*x*+3/2, −*y*, *z*−1/2; (ii) −*x*+1/2, −*y*, *z*−1/2; (iii) *x*+1, *y*, *z*.
